# Experimental Study on Mechanical Properties of European Oak and Norway Spruce Clear Wood

**DOI:** 10.3390/ma18143257

**Published:** 2025-07-10

**Authors:** Serena Gambarelli, Josipa Bošnjak, Rey Noé Fararoni Platas, Kexin Jin

**Affiliations:** Materials Testing Institute, University of Stuttgart, 70569 Stuttgart, Germany; serena.gambarelli@mpa.uni-stuttgart.de (S.G.); rey-noe.fararoni-platas@mpa.uni-stuttgart.de (R.N.F.P.); st192714@stud.uni-stuttgart.de (K.J.)

**Keywords:** uniaxial behaviour, softwood and hardwood, clear wood, orthotropic behaviour, stress–strain curves, failure mode

## Abstract

The trends in the building industry related to sustainability and environmental footprint make timber structures more appealing than ever. Many challenges in understanding the behaviour of structural timber can be addressed by combining experimental and numerical methods. However, sophisticated numerical tools require a complete description of the behaviour at the material level. Even though there are vast databases on the properties of different species, there are only limited studies on the mechanical response with complete stress–strain curves for all relevant directions. In order to bridge this gap, the present study investigates the mechanical response of European oak (hardwood) and Norway spruce (softwood). Uniaxial tensile and compressive tests were performed on small clear wood specimens. The behaviour was investigated for the direction parallel (longitudinal) and perpendicular to the grain (radial and tangential). Both species exhibit brittle tensile behaviour in all material directions, in contrast to the ductile performance under compression. The tensile strength lies at 70 MPa and 80 MPa for spruce and oak, respectively, whereas both species exhibit a compressive strength of approximately 50 MPa in the longitudinal direction. Due to the narrow range of the investigated density, growth-ring angle and growth-ring width, only a limited effect of these parameters was observed on the tensile behaviour in the longitudinal direction.

## 1. Introduction

Wood is one of the oldest building materials, owing to its availability in nature, low required processing, versatility, light weight, high specific strength, low inertia forces and durability [1,2]. In the modern building industry, wood and various timber products play an important role in tackling the challenges related to sustainability, resource management and environmental impact throughout the complete life cycle, including recycling. On the other hand, wood, as a natural material, exhibits defects such as knots and cracks, which contribute towards high scatter. Along with the fact that material behaviour varies significantly among wood species, this poses a great challenge for researchers and designers alike. Unlike other building materials, wood exhibits orthotropic behaviour with a very different response in the three relevant directions: the grain direction (longitudinal—L), the direction perpendicular to the grain; the the radial direction (R) and the tangential direction (T). Additionally, the fact that the mechanical properties of wood are highly dependent on the moisture content and anatomical structure of wood makes the assessment of material behaviour and structural response even more complex.

Decades of research on the behaviour of wood and timber yielded modern codes and guidelines to simplify the design process, whereby appropriate safety concepts were put in place to assure structural safety. Modern applications of wood in the building sector, with increasing requirements for performance, call for a deeper understanding of the mechanical response as well as the interactions of the physical and mechanical properties. Numerical methods can offer a substantial benefit compared to often employed empirical methods, which may not be valid beyond the underlining experimental basis. One of the main prerequisites for a realistic and reliable numerical analysis is the understanding of the uniaxial behaviour at a material level (i.e., on clear wood with minimal defects). Sophisticated numerical tools require a complete description of the stress–strain response for all relevant directions i.e., a reliable generalized material model that accounts for nonlinearity. Such a model is essential for the non-linear analysis of EWPs and more complex timber systems involving damage initiation and development (cracking). This aspect is fundamental, for example, when investigating moisture-induced cracking problems, which are relevant for the durability of wood and timber.

The majority of existing research studies on the mechanical behaviour of wood focused primarily on properties such as bending strength and bending elasticity modulus, as these are typically required for design purposes and more practical to perform [3,4,5,6]. Furthermore, material is often described only in terms of strength, without providing the complete stress–strain curves [7,8,9]. Due to a lack of a universally accepted testing methods with respect to specimen size and moisture content, the researchers used different geometries (sizes) and moisture conditions to test material properties. For example, various specimen geometries and sizes are used for the investigation of tensile behaviour [10,11,12]. Similarly, some authors considered only extremes of moisture content without relating the behaviour to ambient conditions, i.e., moisture content of 12% [13]. These differences in geometry and moisture content can make the direct comparison of results difficult.

The focus of the present study is to provide a complete description of the material behaviour of clear wood for Norway spruce (softwood) and European oak (hardwood). The objective is to better understand the mechanical performance as well as to provide an experimental basis for the validation of numerical tools for wood. Uniaxial tension and compression in all three relevant directions (L, R and T) were experimentally studied and stress–strain curves and failure modes are reported. After a literature review in Section 2, materials and methods are presented in Section 3. The results of the study are discussed in Section 4. Section 5 highlights the main conclusions of the study.

## 2. Literature Review

The focus of the present literature study is on Norway spruce (*Picea abies*) as softwood and European oak (*Quercus robur*) as hardwood. Norway spruce is one of the most widely used softwoods (evergreens) in Europe. Its suitability for glued laminates (glulam) and other engineered wood products (laminated veneer lumber—LVL, cross-laminated timber—CLT, etc.) contributed substantially to its popularity in modern timber design.

An overview of relevant research studies on spruce clear wood is provided in Table 1 for uniaxial compression and Table 2 for uniaxial tension. It is worth mentioning that only literature studies on Norway spruce (clear wood) tested at ca. 12% of moisture content were selected and summarized for possible correlation with the present study. Some relevant literature studies are not included due to significantly different and/or not quantified moisture content. For example, ref. [13] investigated the influence of strain rate on the stress–strain response of wood in three material directions under uniaxial compression for three states of moisture content (oven-dry, fiber saturated and fully saturated). An increase in moisture content lead to a degradation of compressive strength and, partially, to change in the shape of the stress–strain curves in the post-peak region. Moreover, typical strength ranges or values found in textbooks on wood (such as [1,3]) are not included due to missing references to the original work.

It can be seen (Table 1 and Table 2) that a large variety of geometries and dimensions of the tested samples was investigated over the years, based on the different norms and standards. Some of the research studies focused on elastic and compliance orthotropic parameters [14,15] and others focused on strength values and scatter ranges while investigating various influencing parameters [7,8,9]. The majority of testing under uniaxial tension and compression was performed in the longitudinal direction, whereas very limited studies provided complete stress–strain curves in the three material directions (L, R and T). The influence of size [16,17] as well as different defects [16] was also primarily investigated in the longitudinal direction.

**Figure 1:**
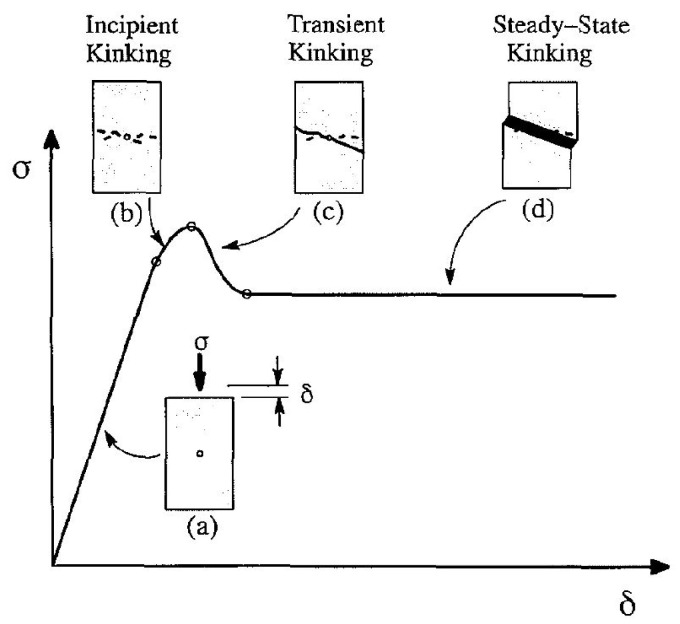
Idealized stress–strain curve and development of kinking (taken from [18]): (**a**) compression test; (**b**) incipient kinking; (**c**) transient kinking; (**d**) steady-state kinking.

**Figure 2:**
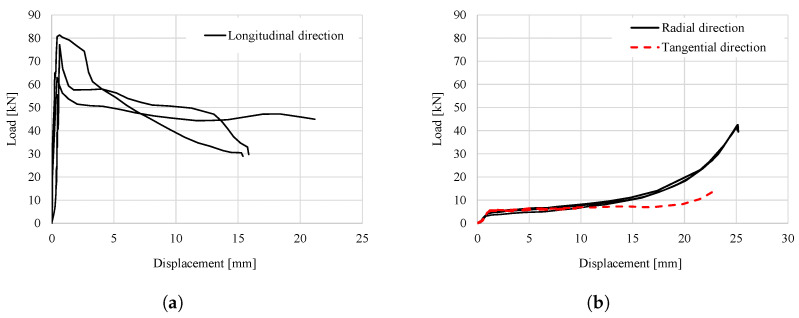
Force–displacement curves for uniaxial compression: (**a**) longitudinal direction; (**b**) radial and tangential directions (reproduced from [10]).

The compressive behaviour of Norway spruce has been largely investigated over the years [7,8,10,16,17,18,19,20,21,22,23,24,25]. Poulsen [18,19] was among the first to extensively describe the kinking failure of clear wood under uniaxial compression in the longitudinal direction, by using specimens with a small hole in the central region. An idealized curve was elaborated to explain the three main stages of the kinking mechanism, i.e., incipient kinking, transient kinking and steady-state kinking (see Figure 1). Franke [10] investigated the compressive behaviour of Norway spruce in all three relevant directions on cubes with 40 mm side length. The obtained stress-strain curves are shown in Figure 2. Zhong et al. [26] investigated the compressive behaviour of spruce clear wood along the three material directions (L, R and T) on cuboic specimens (20 × 20 × 30 mm^3^). The stubby geometry was chosen to capture the behaviour at large strains. Different failure modes were observed, namely, fibres buckling and collapsing under compression in the longitudinal direction, and fibre slippage and delamination in the radial and tangential directions. The stress–strain curves for Norway spruce in compression obtained by Poulsen [18,19], Franke [10] and Zhong et al. [26] are qualitatively comparable, in spite of differences in specimen geometry.

**Table 1:** Literature studies on spruce clear wood under uniaxial compression.

Ref.	Species	ρ [kg/m^3^]	Geometry W × T × L [mm^3^]	Moisture Content [%]	N. per Dir. [L, R, T]	Output
Poulsen et al.	*Picea abies*	410	Prisms	12	(NS, -, -)	Typical σ-d curves,
(1997) [18]	(Norway spruce)		(14 × 14 × 50)			failure mode
Reiterer et al.	*Picea abies*	400–430	Prisms	12–13	(10, 10, -)	Typical σ-ϵ curves,
(2001) [20]	(Norway spruce)		(10 × 10 × 20)			failure mode
Gindl and Teischinger	*Picea abies*	400	Prisms	12	(47, -, -)	Strength vs. density
(2002) [7]	(Norway spruce)		(12 × 12 × 6)			
Franke	*Picea abies*	415	Cubes	9.9	(5, 3, 3)	σ-ϵ curves,
(2008) [10]	(Norway spruce)		(40 × 40 × 40)			Strength, E-modulus
Zhong et al. (2014) [26]	*Picea abies* (Norway spruce)	413	Cuboids (20 × 20 × 30)	12.7	(3, 5, 4)	σ-ϵ curves, failure mode
Zauner and Niemz	*Picea abies*	352–475	Dog-bone	EMC	(8-12, -, -)	σ-ϵ curves, strength
(2014) [17]	(Norway spruce)		(different sizes)			
Aicher et al.	*Picea abies*	440	Prisms	13.9	(42, -, -)	Strength vs. MC,
(2016) [23]	(Norway spruce)		(20 × 20 × 120)			typical σ-ϵ curves
			Prisms		(42, -, -)	
			(30 × 30 × 60)			
Bieniasz et al.	*Picea abies*	422	Prisms	12	(NS, -, -)	Strength, E-modulus
(2017) [8]	(Norway spruce)		(20 × 20 × 30)			
Akter and Bader	*Picea abies*	499	Dog-bone	12	(-, 2, 2)	σ-ϵ curves
(2020) [24]	(Norway spruce)		(50 × 60 × 20)			
		492	Quadratic		(-, 2, 2)	
			(50 × 50 × 20)			
Šmídová et al. (2022) [11]	*Picea abies* (Norway spruce)	463	Prisms (45 × 70 × 90)	8	(5, -, -)	L-d curves, failure mode
Jakob and G.-Altmutter	*Picea abies*	470	Prisms	10	(-, 5, 5)	σ-ϵ curves
(2023) [25]	(Norway spruce)		(15 × 15 × 6)			
Li et al.	*Picea abies*	-	Prisms	12	(20, -, -)	L-d curves, strength
(2024) [16]	(Norway spruce)		(40 × 65 × DS)			failure mode

ρ is the density at 20 °C and 65% relative humidity (RH); EMC is the equilibrium moisture content (MC).

The uniaxial tensile behaviour of Norway spruce has received less attention than compression, possibly because bending tests are commonly used to measure tensile strength. Poulsen [19] investigated Norway spruce under uniaxial tension in the tangential direction on dog-bone specimens and reported stress–strain curves. Several authors [9,10,11] investigated the tensile behaviour in the longitudinal direction on dog-bone specimens with different geometries. The only studies reporting tensile behavior in all three directions (L, R and T) were focused on elastic properties, without providing stress–strain curves [14,15].

European oak (*Quercus robur*) is a deciduous species native to Europe and parts of western Asia. It is widely used as structural timber, as well as for cladding and flooring. In comparison with spruce, there are only a few studies focusing on the behaviour of oak clear wood [27,28,29,30,31]. An overview of these studies is provided in Table 3. Ljungdahl et al. [27], Volkmer et al. [29] and Ozyhar et al. [30] investigated the compressive behavior in all three material directions (L, R, T). However, only Ljungdahl et al. [27] provided complete stress–strain curves. Uniaxial tensile behavior was mainly investigated in the longitudinal direction [28,31]. Only Volkmer et al. [29] reported elastic and strength values for both tension and compression in the three material directions.

**Table 2:** Literature studies on spruce clear wood under uniaxial tension.

Ref.	Species	ρ [kg/m^3^]	Geometry W × T × L [mm^3^]	Moisture Content [%]	N. per Dir. [L, R, T]	Output
Poulsen	*Picea abies*	-	Dog-bone	EMC	(-, -, 12)	σ-ϵ curves
(1998) [19]	(Norway spruce)		(20 × 20 × 20)			
Yoshihara and Ohta (2000) [9]	Picea sichtensis (Sitka spruce)	-	Dog-bone (2 × 10 × 140)	EMC	(5, 5, -)	Strength vs. off-axis angle
Dill-Langer et al.	*Picea abies*	500	Dog-bone	12	(-, 4, 5)	Typical σ-ϵ curves,
(2002) [32]	(Norway spruce)		(5 × 10 × 60)			failure mode
Miyauchi and Murata	*Picea* sp.	552	Dog-bone	10.4	(-, 22, -)	Strength, failure mode,
(2007) [12]	(Spruce)		(10 × 20 × 150)			typical σ-ϵ curves
Keunecke et al.	*Picea abies*	470	Dog-bone	EMC	(15, 15, 15)	E-modulus,
(2008) [14]	(Norway spruce)		(14 × 14 × 95)			Poisson’s ratio
Franke	*Picea abies*	484	Dog-bone	12.4	(40, -, -)	σ-ϵ curves,
(2008) [10]	(Norway spruce)		(2 × 20 × 475)			E-modulus
Kumpenza et al.	*Picea abies*	465	Dog-bone	12	(20, 24, 23)	E-modulus,
(2018) [15]	(Norway spruce)		(20 × 6 × 120)			Poisson’s ratio
Šmídová et al. (2022) [11]	*Picea abies* (Norway spruce)	463	Prisms (4 × 25 × 250)	8	(10, -, -)	L-d curves, failure mode

ρ is the density at 20 °C and 65% relative humidity (RH); EMC is the equilibrium moisture content (MC).

## 3. Materials and Methods

Two wood types were experimentally investigated in this study, namely, Norway spruce (*Picea abies*) as softwood and European oak (*Quercus robur*) as hardwood. The aim was to provide for each wood a complete database in terms of stress–strain curves and failure modes in the three material directions, i.e., longitudinal (L), radial (R) and tangential (T).

All specimens for testing were cut from solid timber beams obtained from Hermann Metzger GmbH & Co. in Vaihingen an der Enz, Germany. The original dimensions of the timber beams are 2500×140×140 mm^3^ for spruce and 2500×120×120 mm^3^ for oak. In order to obtain samples with clear orientation of the growth rings in the radial (R) and tangential (T) directions, only the beams extracted from the most external parts of the tree trunk were selected by the authors. It is worth mentioning that only a few beams available from the company could satisfy this criterion.

**Figure 3:**
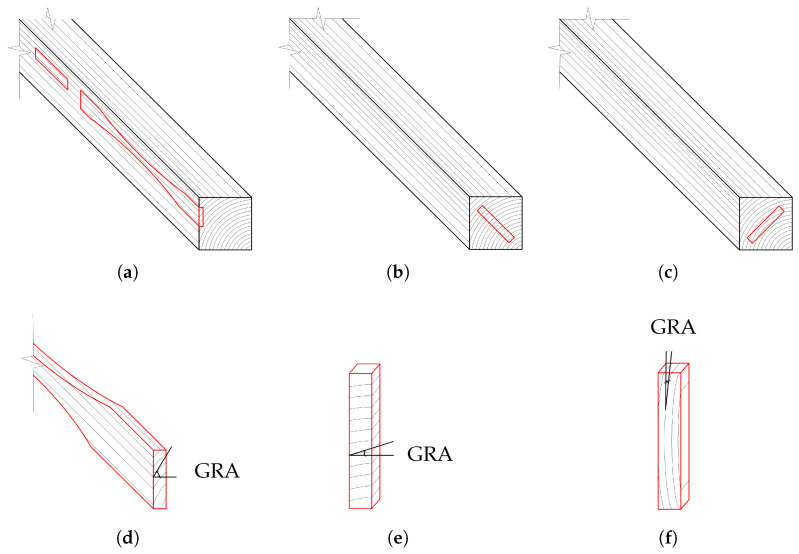
Cutting scheme of the samples in L (**a**), R (**b**) and T (**c**) directions. Qualitative representation of the growth-ring angle (GRA) in L (**d**), R (**e**) and T (**f**) directions.

The beams were first cut longitudinally to obtain slices with the desired thickness of the different specimen types. Thereafter, the final specimens were cut with predefined orientation of the rings (R and T directions) and grains (L direction). During cutting, an effort was made to obtain clear wood specimens (almost free of defects). Figure 3 shows the cutting scheme of the test samples in the three material directions.

Uniaxial tensile and compressive tests on the two wood species were performed in the three material directions (L, R and T) for a total of 12 investigated cases. Between 7 and 16 specimens were tested for each case. A summary of the performed tests for spruce and oak is reported in Table 4. In each abbreviation, the first letter indicates the wood specie (spruce or oak), the second one the loading type (compression or tension) and the last one the loading direction (L, R or T), i.e., S-TL means Spruce under Tension in the Longitudinal direction.

**Figure 4:**
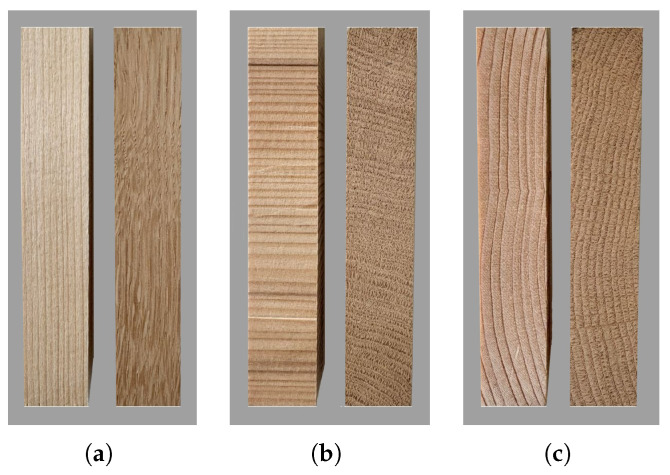
Oak and spruce test specimens for the uniaxial compressive test: (**a**) longitudinal direction; (**b**) radial direction; (**c**) tangential direction.

Dog-bone specimens were used only for the uniaxial tensile test in the longitudinal direction (L), according to DIN 52377 [33]. In the specified dimensions (50×10×443 mm^3^), the 10 mm thickness refers to the minimum dimension in the central region of the specimen. Square prisms (20×20×120 mm^3^) were used for all other tests, according to EN 408 [34]. Representative test specimens are shown in Figure 4 for the two woods. It is visible that the orientation of the growth rings mainly deviates from the tangential direction for both spruce and oak (Figure 4c). The same applies to the radial direction for oak (Figure 4b). A slight deviation in the grains is observed for the longitudinal direction (Figure 4a). These aspects may affect the measured strength, as will be discussed in the Section 4.

All samples were conditioned in a climatic room at 20 °C and 65% RH for one month. Prior and after testing, several parameters were measured in all specimens, namely, density at 20 °C and 65% RH (ρ), oven-dry density, moisture content (MC), growth-ring width (GRW) and growth-ring angle (GRA). A summary of the measured parameters is reported in Table 5 for spruce and Table 6 for oak, as mean values and the corresponding coefficient of variation (CoV). The moisture content was calculated according to the oven-dry method [35]. The GRA measurement in the three material directions is qualitatively shown in Figure 3. For each specimen, the GRA was also obtained as average of three measurements. The GRW and GRA were not measured in the specimens under compression in the longitudinal direction (S-CL and O-CL). The reason is that these have no influence on the material response. A relatively high scatter (CoV) in the GRA measurement is observed in Table 5 and Table 6. This confirms that in most R and T specimens, the actual inclination of the rings deviates from the ideal case (straight rings parallel to the loading direction). As documented in DIN 52188 [36], the recommended GRA for S-TL and O-TL should be 90° according to Figure 3. However, this condition could not always be satisfied. Therefore, the influence of the GRA on the measured strength was further investigated and the results are shown in Section 4.

**Figure 5:**
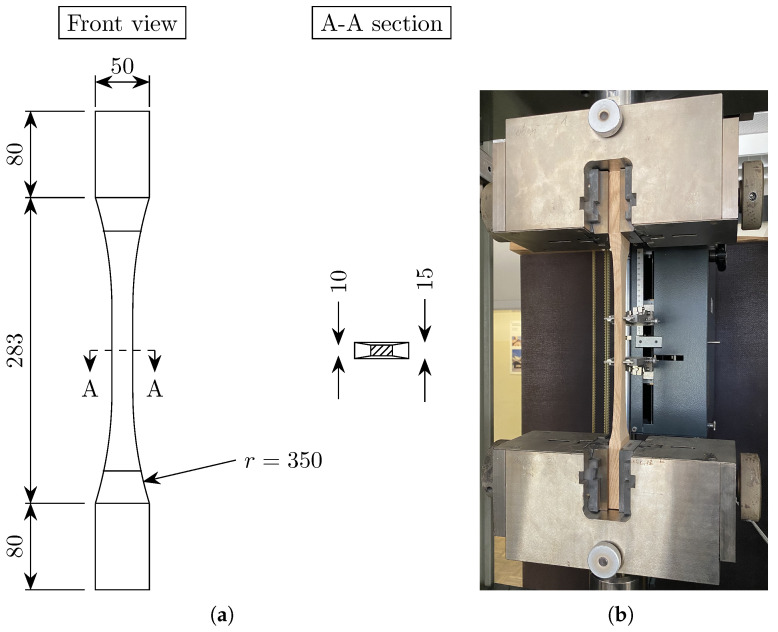
(**a**) Geometry of the specimens for uniaxial tensile test in longitudinal direction (dimensions in mm); (**b**) test setup for uniaxial tensile test in longitudinal direction.

The mechanical tests were carried out with an electro-mechanical testing machine from the company ZwickRoell GmbH & Co. The geometry of the dog-bone specimen for tensile tests in the longitudinal direction is provided in DIN 52188 [36]. However, the specimen configuration mentioned by the norm requires a specific gripping system for the application of the tensile load, which was not available at the time of testing. Therefore, the dog-bone specimen was chosen as specified in the standard for plywood (DIN 52377 [33]); see Figure 5. The length of the gripping parts was slightly increased with respect to the norm (from 63 to 80 mm). This was performed in order to increase the gripping area and prevent local compressive damage. The tensile failure in the dog-bone specimen acc. to DIN 52377 [33] is induced in the central region where the section is reduced to a width of 20 mm (see front view in Figure 5a). However, preliminary tests showed that a double constriction (minimum thickness of 10 mm) was necessary for this purpose (see side view in Figure 5a). Figure 5b shows the test setup for the oak specimen. A length of 80 mm was needed on each side of the specimen to properly fix the sample to the gripping system of the machine. An electronic strain extensometer (LD—DD1) with a gauge length of 60 mm was used to measure the vertical strains in the central region of each specimen (see Figure 5b). A displacement-controlled test was carried out with a rate of 3 mm/min for spruce and 2.5 mm/min for oak, up to a load level of 10 kN. At this point, the extensometer was removed and the loading was resumed at a displacement rate of 9 mm/min until failure.

**Figure 6:**
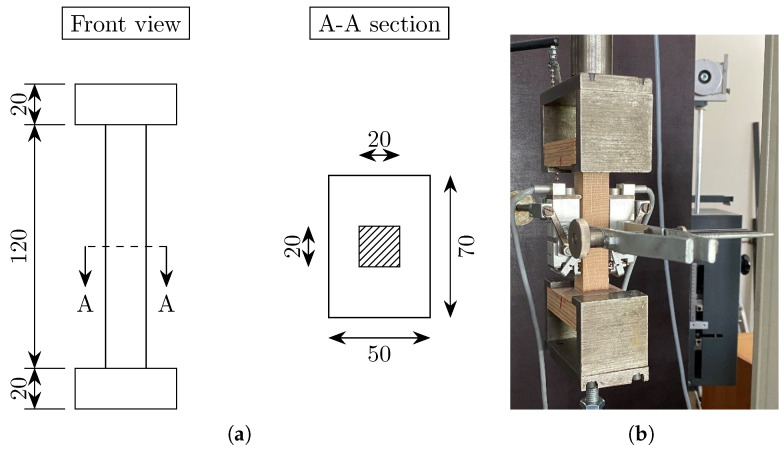
(**a**) Geometry of the specimens for uniaxial tensile test in radial and tangential directions (dimensions in mm); (**b**) test setup for uniaxial tensile test in radial direction.

As mentioned above, the uniaxial tensile tests in the radial (R) and tangential (T) directions were performed for both woods on square prisms with dimensions 20×20×120 mm^3^ (width × thickness × length) according to EN 408 [34]. Based on the norm (specific for timber), the length of the specimen (120 mm) should be at least six times that of the smaller cross-sectional dimension (20 mm). The specimens were glued with a two-component polyurethane adhesive to LVL (laminated veneer lumber) plates (20×50×70 mm^3^) properly fixed in the loading machine with grips. The geometry of the test specimens is shown in Figure 6a. The test setup for oak in the tangential direction is shown in Figure 6b. A gauge length of 50 mm was chosen in this case. A displacement-controlled test was carried out with a rate of 0.8–1.3 mm/min for spruce and oak in the radial direction. The loading rate was chosen such to reach the peak load within 1–2 min. In all tensile tests, the extensometer was removed at ca. 80% of the peak load to avoid possible damage. Therefore, the obtained stress–strain curves (see Section 4) were linearly extended with dashed lines up to the peak.

**Figure 7:**
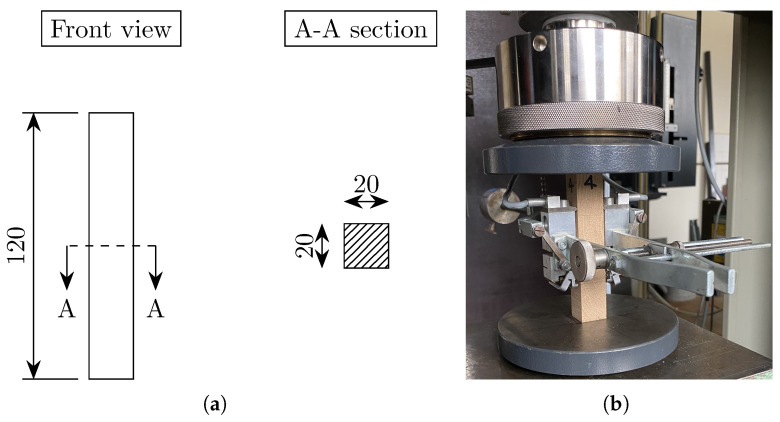
(**a**) Geometry of the specimens for compressive tests in longitudinal, radial and tangential directions (dimensions in mm); (**b**) test setup for compressive tests on oak in tangential direction.

The uniaxial compressive test in the three directions (L, R and T) was carried out on square prisms (20×20×120 mm^3^), according to EN 408 (Table 4). A higher length with respect to that specified in ISO 13061-17 [37] (80 mm) and DIN 52185 [38] (60 mm) was chosen in order to fit the available extensometer for the strain measurements in the central area of each specimen. The section is the same as proposed in the two standards. The test setup is shown in Figure 7. The specimen was placed between the steel plates of the loading machine for the application of the compressive load. The gauge length of the extensometer was set equal to 50 mm.

A displacement-controlled test was carried out with a rate of 0.8 mm/min for both spruce and oak in the longitudinal, radial and tangential directions.

## 4. Results and Discussion

### 4.1. Uniaxial Tensile Test in the Longitudinal Direction

**Figure 8:**
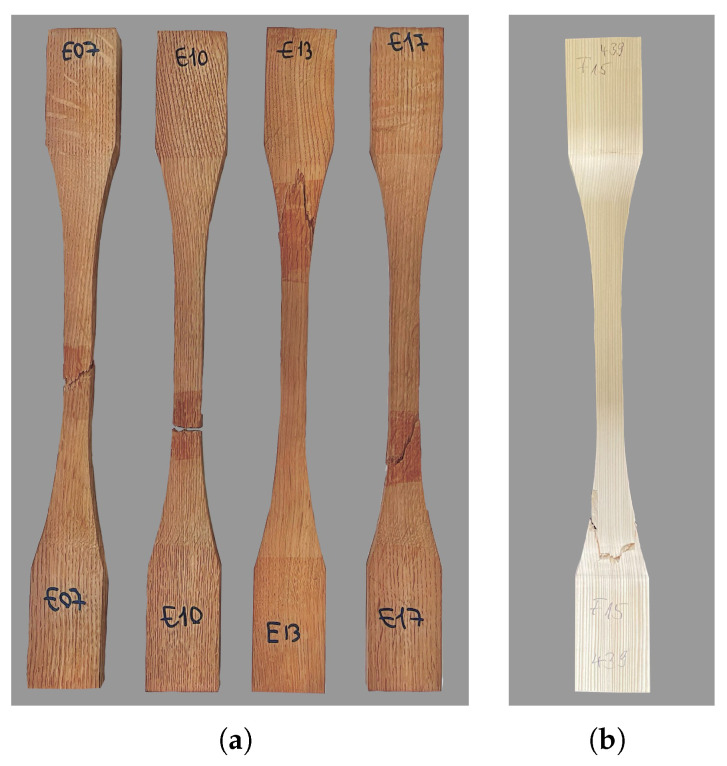
Typical failure modes under tension in the longitudinal direction. (**a**) Oak: combined tension and shear, brittle tension, splinter and shear. (**b**) Spruce: splinter.

A summary of the test results for oak and spruce under uniaxial tension in the longitudinal direction is shown in Table 7. The table reports, for each wood type, the observed failure mode, as well as the mean strength and stiffness (with corresponding CoV), measured on 16 specimens. As documented in [1], typical tensile failure modes can be classified as splinter, combined tension and shear, shear and brittle tension. Regarding oak, out of 16 tests, 4 specimens failed in splinter, 10 in brittle tension, 1 in shear and 1 in combined tension and shear. A clear comparison between the different failure modes observed in oak is shown in Figure 8. For spruce, most specimens (11) failed in splinter.

**Figure 9:**
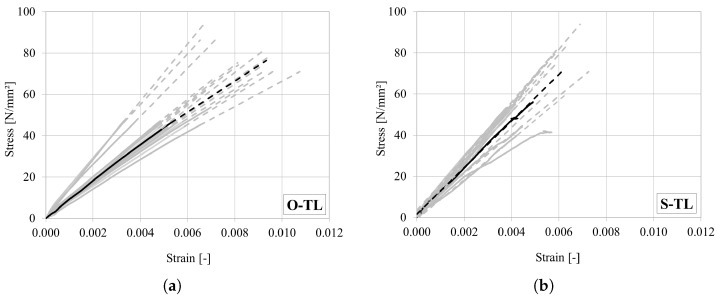
Stress–strain curves under tension in the longitudinal direction for (**a**) oak and (**b**) spruce. (Dashed lines represent linear extension up to the peak. Representative curves are marked in bold).

All stress–strain curves, with strain taken from the extensometer, are shown in Figure 9a for oak and Figure 9b for spruce. It can be seen that the curves are almost linear elastic up to the peak. Due to the very brittle behaviour of wood under tension, the post-peak phase was not captured in the tests. A representative curve for each wood type is marked in bold. Strength and stiffness were evaluated for each sample, based on the ratio between stress and strain, which measured between 10% and 30% of the peak load (see [39]). The average strength measured in spruce is slightly lower than that reported in the available literature data [1,40], while the stiffness is consistent with previous studies summarized in [1]. In the case of oak, both mean strength and stiffness (Table 7) are in good agreement with the values reported in Volkmer et al. [29].

The influence of oven-dry density, growth-ring thickness (GRW) and growth-ring angle (GRA) was also investigated in correlation to the obtained stress–strain curves and the observed failure mode. These aspects are discussed in Section 4.7.

### 4.2. Uniaxial Tensile Test in the Radial Direction

**Figure 10:**
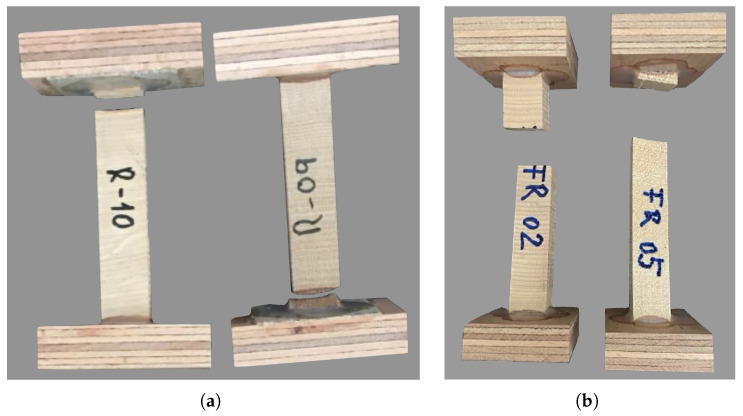
Failure mode of (**a**) oak and (**b**) spruce under tension in the radial direction.

A summary of the results for the uniaxial tensile test in the radial direction is reported in Table 8. All oak specimens failed in brittle tension close to the adhesive (see Figure 10a). In the case of spruce, only specimen 2 failed in the central part and all others failed close to the adhesive (Figure 10b).

**Figure 11:**
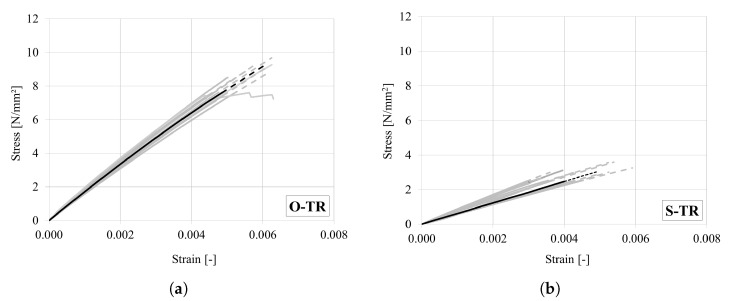
Stress–strain curves under tension in the radial direction for (**a**) oak and (**b**) spruce. (Dashed lines represent linear extension up to the peak. Representative curves are marked in bold).

All stress–strain curves are shown in Figure 11a for oak and Figure 11b for spruce. The same as for the longitudinal case, an elastic-brittle behaviour was observed in all tests. The average strength and stiffness values for both wood species (Table 8) are consistent with those reported in the literature [1,29] and the corresponding scatter is not significant.

### 4.3. Uniaxial Tensile Test in the Tangential Direction

**Figure 12:**
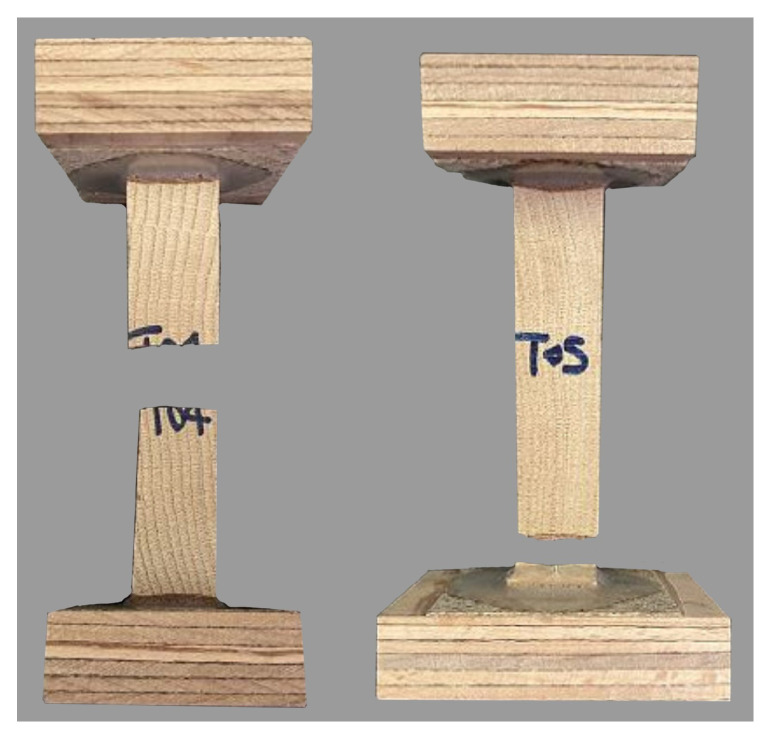
Failure mode for oak under tension in the tangential direction.

The results of the tensile test in the tangential direction are summarized in Table 9 for both wood species. The same as for the radial direction, all tested specimens failed in brittle tension. In the case of oak, 7 specimens underwent tension failure in the central region, while the remaining 4 failed close to the adhesive. Differently, most of the spruce specimens (10) failed close to the adhesive and the remaining 2 failed in the central region. Figure 12 shows the typical tensile failure observed in oak.

**Figure 13:**
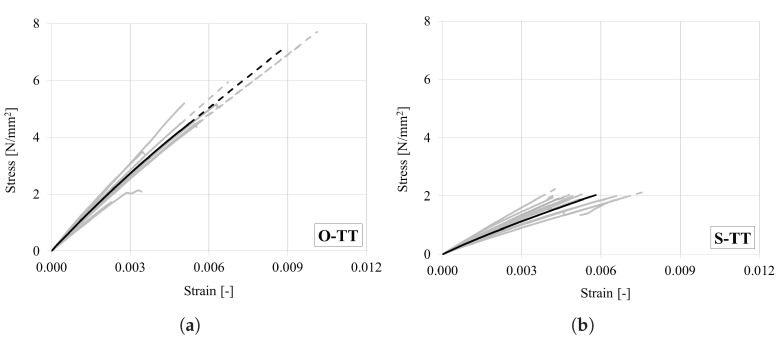
Stress–strain curves under tension in the tangential direction for (**a**) oak and (**b**) spruce. (Dashed lines represent linear extension up to the peak. Representative curves are marked in bold).

All stress–strain curves are shown in Figure 13a for oak and in Figure 13b for spruce. The curves exhibit linear elastic behavior up to the peak. After the strength is reached, a sudden drop in the curves is observed due to brittle failure. The mean strength measured in oak (Table 9) is slightly lower than that reported in the available literature data [1,29]. A broad range of strength values (from 1.7 to 7.7 MPa) was observed in the test, with consequently relatively high CoV (Table 9). The mean stiffness obtained for oak (see Table 9) is in good agreement with the values reported in the available literature studies [1,29]. In case of spruce, both mean strength and stiffness are consistent with the results reported in the literature [1].

### 4.4. Uniaxial Compressive Test in the Longitudinal Direction

**Figure 14:**
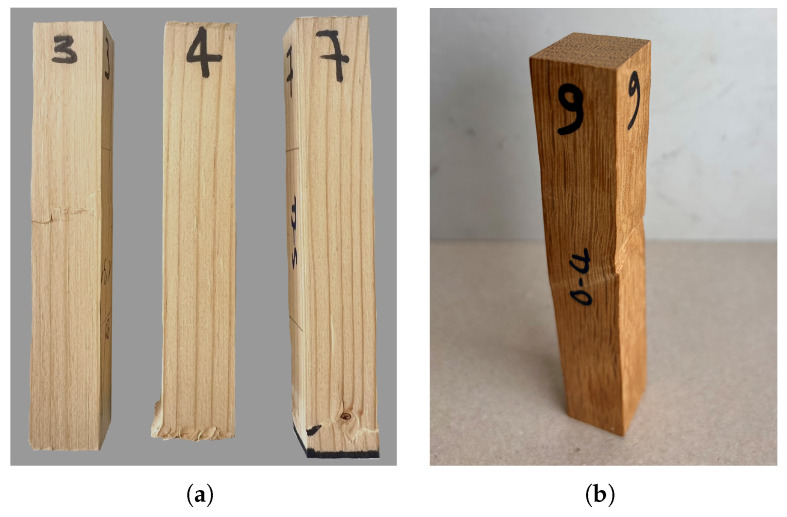
Typical failure modes under compression in the longitudinal direction. (**a**) Spruce: shear (3), end rolling (4) and crushing (7). (**b**) Oak: shear band kinking.

A summary of the test results for both wood types is shown in Table 10. All oak specimens failed due to shearing. It is worth mentioning that specimens 3, 5 and 8 each have two knots and specimen 7 has one big knot in the region of the failure; therefore, these were excluded from evaluation. In the case of spruce, five specimens underwent shear failure, one failed due to crushing and three failed due to end rolling (see Figure 14a). Specimen 7 was excluded from evaluation as it exhibited two knots. The obtained mean strength, stiffness and CoV (Table 10) are, for both woods, in good agreement with the literature data [1,29].

As explained in [18], the shear behaviour of wood under compression is characterized by three stages, namely, incipient, transient and steady-state kinking. In the first stage, a local bending of the fibres is observed. Thereafter, the fibres are rotated with respect to the original orientation. At high deformations, a kink band is formed, which is inclined (shear band) in the L-T plane and orthogonal to the fibre direction in the L-R plane [16] (see Figure 14b).

**Figure 15:**
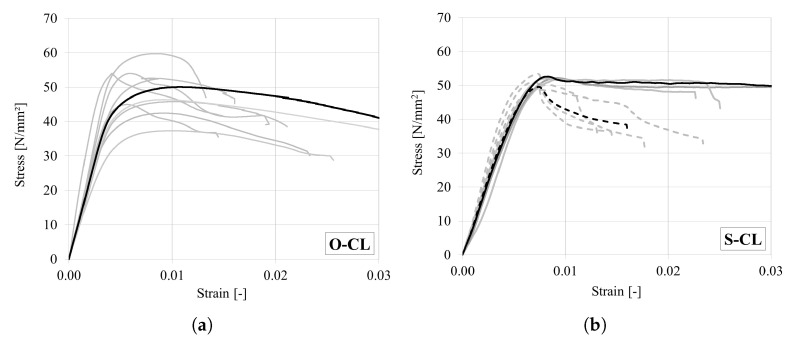
Stress–strain curves under compression in the longitudinal direction for (**a**) oak and (**b**) spruce. (Representative curves are marked in bold. Note that in case of spruce the full lines represent end rolling failure and dashed lines shear failure).

The stress–strain curves obtained for oak and spruce are shown in Figure 15. It can be seen that in contrast to the very brittle tensile failure, wood exhibits a ductile behaviour under compression. The curves are almost linear elastic, with some non-linearity just before the peak (incipient kinking). Thereafter, the stress slightly reduces (transient kinking) and then either stabilizes or further reduces at a slower rate (steady-state kinking).

### 4.5. Uniaxial Compressive Test in the Radial Direction

**Figure 16:**
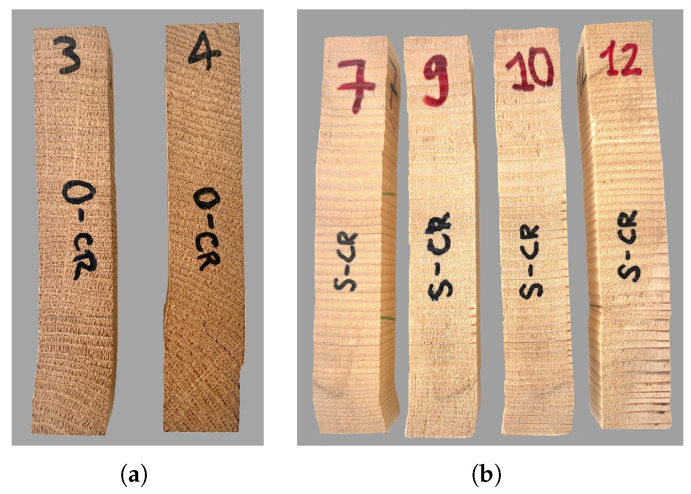
Typical failure modes under compression in the radial direction. (**a**) Oak: shear (4) and cell buckling (3). (**b**) Spruce: plastification due to cell buckling along the specimen height.

The main results of the test are summarized in Table 11. In the case of oak, specimen 4 was excluded from evaluation, as it experienced shear failure due to high inclination of the growth rings (see Figure 16a). All other oak and spruce specimens with clear radial direction failed due to cell buckling, at the level of the growth rings. As shown in previous studies [1,25,41], the earlywood, with lower density, collapses first due to buckling, and then the latewood collapses. This plastification takes place in different locations over the length. Due to the high slenderness of the specimen (h/a = 6), a structural buckling is observed as final failure, after which the test was stopped (Figure 16). Theoretically, with increasing compressive strains (above 40%), a solidification of the material occurs with a significant increase in the material strength, which is proportional to the increasing density [1,26]. This behaviour, observed at very high strain levels, was not investigated in the present study.

**Figure 17:**
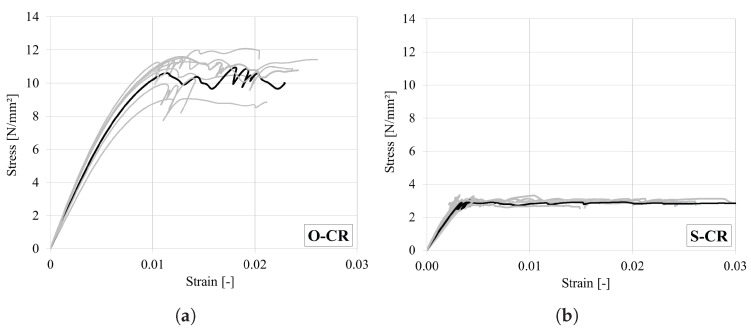
Stress–strain curves under compression in the radial direction for (**a**) oak and (**b**) spruce. (Representative curves are marked in bold).

The obtained stress–strain curves for oak and spruce are shown in Figure 17. It can be seen that both woods have ductile compressive behaviour. The mean strength and stiffness values are consistent with those reported in the literature [1,29]. The strength and stiffness of oak are significantly higher than those of spruce.

### 4.6. Uniaxial Compressive Test in the Tangential Direction

**Figure 18:**
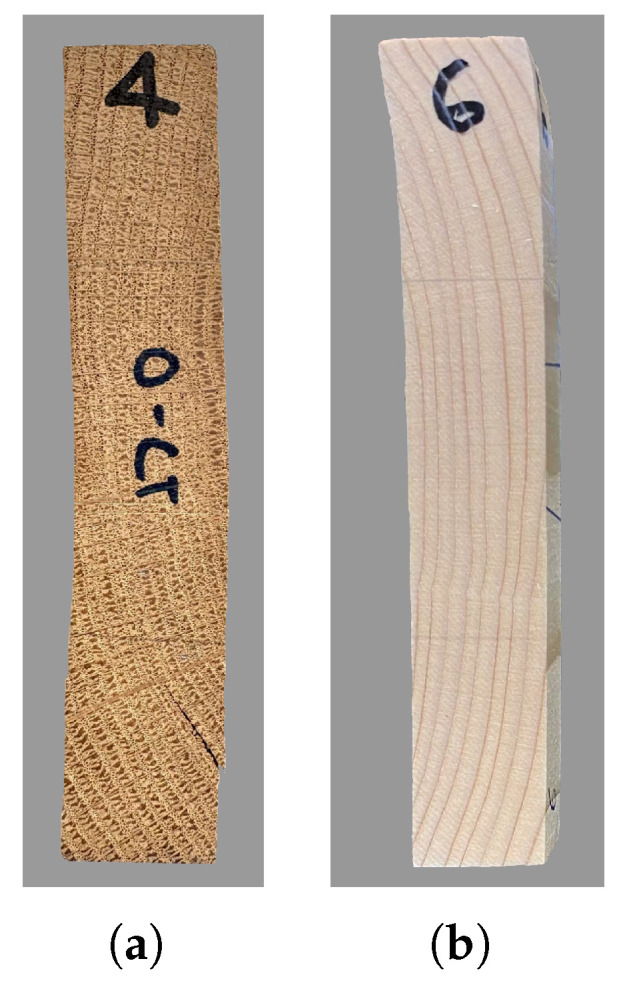
Typical failure under compression in the tangential direction for (**a**) oak and (**b**) spruce.

**Figure 19:**
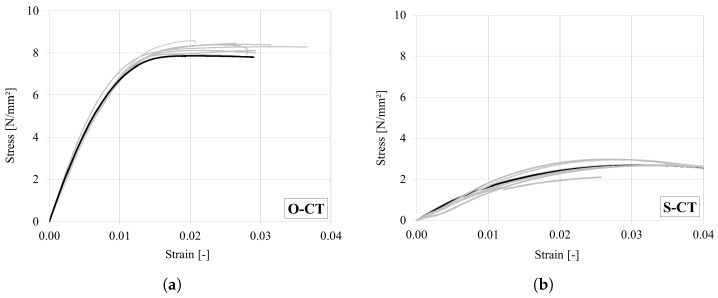
Stress–strain curves under compression in the tangential direction for (**a**) oak and (**b**) spruce. (Representative curves are marked in bold).

The test results are summarized in Table 12. In the case of oak, seven specimens failed due to buckling caused by the inevitable deviation of the growth rings from the ideal tangential direction. Slender specimen geometry contributed towards buckling. The remaining three specimens exhibited shear failure due to the high inclination of the growth rings (ca. 45°) close to the loading plates (Figure 18a). All spruce specimens failed due to buckling (Figure 18b). The obtained stress–strain curves for spruce and oak are shown in Figure 19. The same as for the radial case, a ductile compressive behavior is observed in the tangential direction. The mean strength, stiffness and CoV obtained for both woods are consistent with results reported in [1,29].

### 4.7. Influence of Relevant Parameters on the Strength and Elasticity Modulus

**Figure 20:**
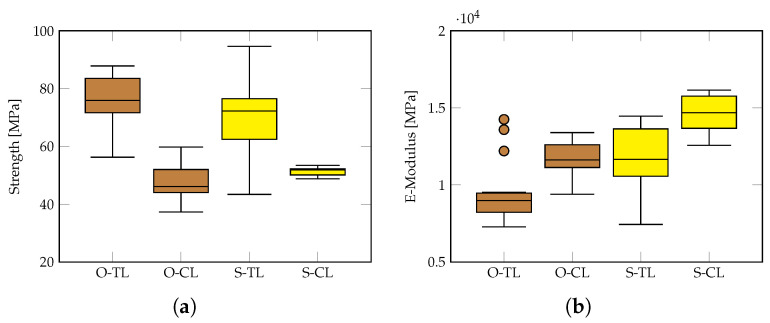
Summary of results for oak and spruce in the longitudinal direction: tensile and compressive (**a**) strength and (**b**) E-Modulus. Note that brown colour refers to oak and yellow to spruce.

A summary of the results for oak and spruce in the longitudinal direction is shown in Figure 20. It can be seen that for both species, the tensile strength and stiffness exhibit higher scatter than in the case of compression. Also, it is interesting to observe that the E-modulus in compression is higher than in tension for both spruce and oak.

**Figure 21:**
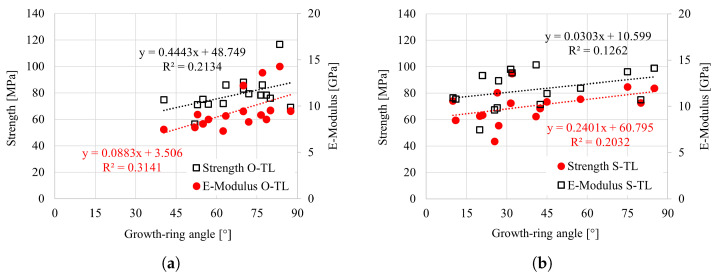
Strength vs. growth-ring angle (GRA) for (**a**) oak and (**b**) spruce under tension in the longitudinal direction.

**Figure 22:**
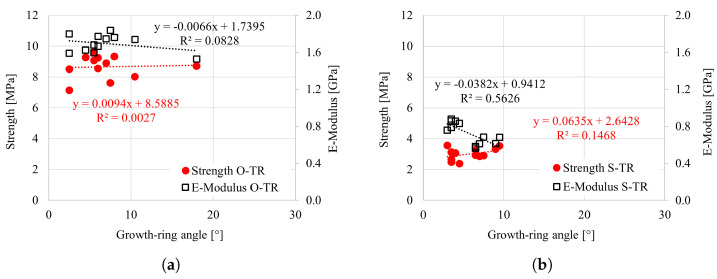
Strength vs. growth-ring angle (GRA) for (**a**) oak and (**b**) spruce under tension in the radial direction.

**Figure 23:**
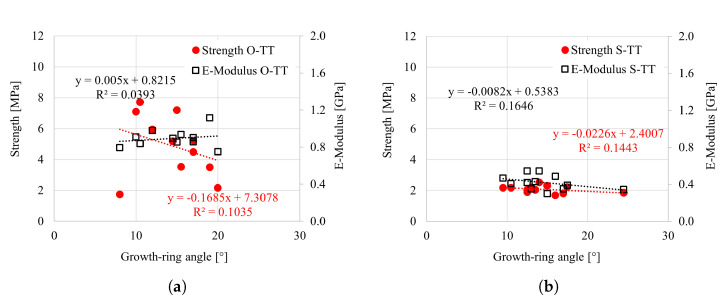
Strength vs. growth-ring angle (GRA) for (**a**) oak and (**b**) spruce under tension in the tangential direction.

The influence of relevant parameters, i.e., growth-ring angle (GRA), growth-ring width (GRW) and oven-dry density, was analyzed for all tested specimens. The effect of the GRA for tensile tests in the longitudinal direction is shown in Figure 21a,b. The focus was primarily on the tensile loading, as the GRA does not impact the response under compression. It is visible that the strength and elasticity modulus increase with increasing inclination of the growth rings. However, the overall impact appears moderate. This is particularly visible in the case of Norway spruce, where a wide range in the GRA (from 10° to 85°) was observed. The effect of the GRA on the strength and elasticity modulus for the radial and tangential directions (under tensile loading) is negligible, particularly considering the overall scatter and relatively narrow range of GRA; see Figure 22 and Figure 23.

The GRW varied between 1.5 mm and 3 mm. Since this range is relatively narrow, no clear trend with respect to the effect on the strength and elasticity modulus could be observed.

**Figure 24:**
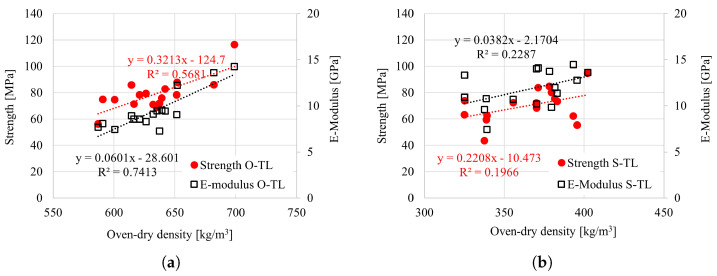
Strength vs. oven-dry density for (**a**) oak and (**b**) spruce under tension in the longitudinal direction.

It was observed in the longitudinal tensile test that the strength and elasticity modulus increase with increasing oven-dry density (Figure 24). It is worth mentioning that the density range was narrow. For the radial and tangential directions, as well as compression in all directions, no clear trend was observed due to a very narrow range of density.

## 5. Conclusions and Outlook

In the present study, the mechanical behaviour of two wood types, i.e., Norway spruce and European oak, was experimentally investigated. Uniaxial tensile and compressive tests were carried out in the three material directions, namely, longitudinal (L), radial (R) and tangential (T). Based on the obtained results, the following can be concluded:

(1) The observed material behaviour is, in general, consistent with existing literature data;

(2) Both spruce and oak exhibit very brittle failure behaviour under tensile load and ductile behaviour under compression;

(3) Strength and stiffness in the longitudinal direction are, for both woods, significantly higher than in the radial and tangential directions;

(4) In most of the analysed cases, the scatter (CoV) in the measured strength and stiffness is not significantly high;

(5) The experimental results provide a complete database, in terms of stress–strain curves and failure mode, which can be used for the calibration and validation of numerical tools.

The authors have implemented the data from the present study in the existing numerical tool [42]. The model is currently being validated with respect to mechanical, thermal and hygral behaviour. The aim is to numerically investigate the complex hygro- and thermo-mechanical coupling in wood and engineered wood products (EWP). A first study in this direction is focused on the mechanical behaviour of a timber (softwood and hardwood) composite connection [43]. 

## Figures and Tables

**Table 3:** Literature studies on oak clear wood.

Ref.	Species	ρ [kg/m^3^]	Geometry W × T × L [mm^3^]	Moisture Content [%]	Load	N. per Dir. [L, R, T]	Output
Ljungdahl et al.	*Quercus robur*	777	Prisms	11.6	UC	(5, 5, 5)	σ-ϵ curves,
(2006) [27]	(European oak)	(50–55% RH)	(10 × 10 × 25)	(50–55% RH)			failure mode
Gustafsson	*Quercus robur*	719	Prisms	5.26	UC	(10, -, -)	σ-ϵ curves
(2010) [28]	(European oak)		(15 × 15 × 45)				
		-	Dog-bone	5.5	UT	(5, -, -)	σ-ϵ curves
			(NS)				
Volkmer et al.	*Quercus robur*	620–670	Prisms	11.0	UC	(10, 10, 10)	Strength, E-modulus,
(2014) [29]	(European oak)		(20 × 20 × 30) (L)				Poisson’s ratio
			(15 × 15 × 45) (R,T)				
			Dog-bone	10.8	UT	(10, 10, 10)	Strength, E-modulus,
			(20 × 6 × 470) (L)				Poisson’s ratio
			(14 × 14 × 95) (R,T)				
Ozyhar et al.	*Quercus robur*	612	Prisms	13.1	UC	(13, 13, 13)	Strength, E-modulus,
(2016) [30]	(European oak)		(15 × 15 × 45)				Poisson’s ratio
Büyüksarı et al.	*Quercus petraea*	-	Prisms	12	UC	(398, -, -)	Compressive strength
(2017) [31]	(Sessile oak)		(20 × 20 × 30)				
			Dog-bone		UT	(309, -, -)	Tensile strength
			(15 × 50 × 400)				

ρ is the density at 20 °C and 65% relative humidity (RH); EMC is the equilibrium moisture content (MC); UC is uniaxial compression; UT is uniaxial tension.

**Table 4:** Overview of the performed tests.

Test	Load	Abbreviation		Geometry	Number of Specimens	
	Direction	Spruce	Oak	[mm^3^]	Spruce	Oak
Tension	L	S-TL	O-TL	dog-bone	16	16
				(50×10×443)		
	R	S-TR	O-TR	Square prism	12	12
				(20×20×120)		
	T	S-TT	O-TT	Square prism	12	11
				(20×20×120)		
Compression	L	S-CL	O-CL	Square prism	9	10
				(20×20×120)		
	R	S-CR	O-CR	Square prism	12	9
				(20×20×120)		
	T	S-CT	O-CT	Square prism	7	10
				(20×20×120)		

**Table 5:** Measurements in spruce specimens. ρ is the density, GRW is the growth-ring width, GRA is the growth-ring angle and MC is the moisture content.

Abbreviation	ρ (CoV)	GRW (CoV)	GRA (CoV)	MC
	[kg/m^3^] ([%])	[mm] ([%])	[°] ([%])	[%]
S-TL	408.3 (6.4)	2.0 (22.1)	39.4 (58.1)	11.7
S-TR	422.7 (2.7)	1.9 (20.8)	5.7 (38.7)	12.9
S-TT	437.3 (3.6)	2.5 (14.4)	14.6 (25.7)	12.3
S-CL	444.5 (5.9)	2.7 (9.4)	-	13.1
S-CR	434.4 (0.9)	2.6 (4.4)	0.45 (1.3)	13.1
S-CT	446.0 (0.9)	2.5 (3.9)	18.8 (11.8)	13.4

**Table 6:** Measurements in oak specimens. ρ is the density, GRW is the growth-ring width, GRA is the growth-ring angle and MC is the moisture content.

Abbreviation	ρ (CoV)	GRW (CoV)	GRA (CoV)	MC
	[kg/m^3^] ([%])	[mm] ([%])	[°] ([%])	[%]
O-TL	707.5 (4.5)	1.8 (13.3)	67.4 (9.3)	11.8
O-TR	698.5 (2.2)	2.3 (7.3)	7.0 (56.8)	13.2
O-TT	690.1 (3.2)	2.1 (5.1)	14.5 (25.4)	14.1
O-CL	727.9 (4.2)	2.2 (6.6)	-	13.6
O-CR	709.7 (2.6)	2.0 (5.7)	11.8 (25.4)	13.6
O-CT	689.0 (3.3)	2.1 (7.8)	19.5 (17.2)	13.6

**Table 7:** Test results for oak and spruce under tension in the longitudinal direction.

Test	N. Spec.	Failure Type	Strength (CoV)	Stiffness (CoV)
			[MPa] ([%])	[MPa] ([%])
O-TL	10	Brittle tension	78.6 (15.7)	9456.7 (21.3)
	4	Splinter		
	1	Shear		
	1	Shear and tension		
S-TL	4	Brittle tension	70.3 (17.4)	11,794.4 (16.6)
	11	Splinter		
	-	Shear		
	1	Shear and tension		

**Table 8:** Test results for oak and spruce under tension in the radial direction.

Test	N. Spec.	Failure Type	Strength (CoV)	Stiffness (CoV)
			[MPa] ([%])	[MPa] ([%])
O-TR	12	Brittle tension	8.7 (8.3)	1693.2 (5.4)
S-TR	12	Brittle tension	3.0 (12.1)	724.6 (15.4)

**Table 9:** Test results for oak and spruce under tension in the radial direction.

Test	N. Spec.	Failure Type	Strength (CoV)	Stiffness (CoV)
			[MPa] ([%])	[MPa] ([%])
O-TT	11	Brittle tension	4.9 (39.5)	893.4 (10.4)
S-TT	12	Brittle tension	2.1 (10.8)	418.5 (18.2)

**Table 10:** Test results for oak and spruce under compression in the longitudinal direction.

Test	N. Spec.	Failure Type	Strength (CoV)	Stiffness (CoV)
			[MPa] ([%])	[MPa] ([%])
O-CL	6	Shearing	49.7 (10.8)	11,679.0 (9.0)
	-	End rolling		
S-CL	5	Shearing	51.5 (2.7)	14,422.0 (8.3)
	3	End rolling		

**Table 11:** Test results for oak and spruce under compression in the radial direction.

Test	N. Spec.	Failure Type	Strength (CoV)	Stiffness (CoV)
			[MPa] ([%])	[MPa] ([%])
O-CR	9	Cell buckling	11.1 (7.0)	1482.0 (8.8)
S-CR	12	Cell buckling	3.2 (3.0)	939.3 (16.2)

**Table 12:** Test results for oak and spruce under compression in the tangential direction.

Test	N. Spec.	Failure Type	Strength (CoV)	Stiffness (CoV)
			[MPa] ([%])	[MPa] ([%])
O-CT	7	Cell buckling	8.2 (2.6)	857.0 (5.7)
	3	Shearing		
S-CT	7	Cell buckling	2.7 (9.9)	370.8 (11.0)
	-	Shearing		

## Data Availability

The datasets presented in this article are not readily available because the data are part of an ongoing study and have not been published yet. Requests to access the datasets should be directed to the corresponding author.
